# Design of a Predictive Recurrence Scoring System in Oral Squamous Cell Carcinoma

**DOI:** 10.3390/jcm15072637

**Published:** 2026-03-30

**Authors:** Cristina Cárdenas-Serres, Fernando Almeida-Parra, Víctor Vega-Barreto, Miguel Ángel Ortega-Nuñez, Julio Acero-Sanz

**Affiliations:** 1 Department of Oral and Maxillofacial Surgery, Ramón y Cajal University Hospital, 28034 Madrid, Spain; victorvegabarreto@gmail.com (V.V.-B.); jacero@salud.madrid.org (J.A.-S.); 2 Ramón y Cajal Institute of Sanitary Research (IRYCIS), 28034 Madrid, Spain; 3 Department of Medicine and Medical Specialities, Faculty of Medicine and Health Sciences, Network Biomedical Research Center for Liver and Digestive Diseases (CIBEREHD), University of Alcalá, 28801 Alcala de Henares, Spain; miguel.angel.ortega92@gmail.com

**Keywords:** oral squamous cell carcinoma, locoregional recurrence, prognostic model, nomogram, recurrence risk score, survival analysis

## Abstract

**Introduction**: Oral squamous cell carcinoma (OSCC) is a frequent head and neck malignancy with high morbidity and mortality. Locoregional recurrence is the main determinant of long-term prognosis, yet reliable predictive models remain limited. This study aimed to identify clinicopathological predictors of recurrence and to develop a nomogram-based recurrence risk score (RRS) for patient stratification. **Materials and Methods**: A retrospective study was conducted including 332 patients with histologically confirmed OSCC treated surgically between 2012 and 2022. Clinical and pathological variables were analyzed. Statistical analyses included Chi-squared tests, Cox regression, Kaplan–Meier survival analysis, and multivariate modeling to construct a predictive nomogram and derive the RRS. **Results:** Close or involved surgical margins were observed in 33.4% of patients, and 66.9% presented moderately or poorly differentiated tumors. Locoregional recurrence occurred in 34.6% of cases, mainly within 24 months. Close margins and poor differentiation were independent predictors of recurrence (*p* < 0.005). The RRS effectively stratified patients into low-, intermediate-, and high-risk groups, with recurrence rates of 21.6%, 50.6%, and 82.1%, respectively (*p* < 0.001). **Conclusions**: The RRS is a practical tool for predicting OSCC recurrence and may support personalized follow-up and adjuvant treatment strategies. Prospective validation is warranted.

## 1. Introduction

Oral squamous cell carcinoma (OSCC) represents one of the most common malignancies and the most common subtype of head and neck cancer. Globally, OSCC represents a significant health burden, with approximately 300,000 newly diagnosed cases [1], and almost 130,000 deaths reported annually [2]. Advanced oral cancer requires multimodality approach with surgical excision ensuring clear margins as the gold standard treatment, while chemotherapy and radiation serve as adjuncts or as primary therapies for unresectable disease. Despite advances in surgical and adjuvant therapies, survival outcomes remain suboptimal, largely due to the high incidence of locoregional recurrence, which occurs in up to 15–40% of cases [3,4,5,6], with significant increasing mortality rates and decreasing overall survival up to 30% when recurrence occurs [7]. Consequently, accurate identification of patients at high risk for recurrence is critical for optimizing follow-up strategies, personalizing adjuvant therapy, and improving overall survival.

Previous studies have identified several clinicopathological factors associated with recurrence, including advanced T and N classification, close or positive resection margins, perineural invasion, extracapsular nodal extension (ENE), and poor histological differentiation [8]. Despite the well-established role of these parameters in survival and guiding therapeutic decisions, there is a lack of standardized tools that integrate clinicopathological factors to predict locoregional recurrence in OSCC.

Nomograms have emerged as valuable prognostic tools in oncology, providing individualized risk estimation by integrating multiple independent variables into a single predictive model [9,10]. However, few studies have developed or validated recurrence-specific nomograms for surgically treated OSCC within European cohorts. The present study aimed to analyze clinicopathological factors associated with locoregional recurrence in a Spanish OSCC cohort and to construct a predictive nomogram-derived recurrence risk score (RRS). This model seeks to stratify patients into clinically meaningful risk groups, facilitating tailored postoperative management and surveillance.

## 2. Materials and Methods

### 2.1. Patient Data Collection

A retrospective data collection was performed at Ramon y Cajal University Hospital, (Madrid, Spain), including 332 patients with histologically confirmed oral squamous cell carcinoma (OSCC) who underwent curative-intent surgery between 2012 and 2022.

Inclusion criteria comprised histologically confirmed OSCC with complete surgical resection and availability of clinicopathological and follow-up data.

Exclusion criteria included unresectable disease, non-surgically treated cases, stage IVC, follow-up < 3 months, early recurrence (<6 weeks), perioperative death, and incomplete data.

Clinicopathological parameters retrieved from medical records included: age, sex, tumor site, T- and N-classification (AJCC 8th edition), histological grade, lymphovascular invasion (LVI), perineural invasion (PNI), extracapsular nodal extension (ENE), surgical margin status, and postoperative radiotherapy. Margins were categorized as: positive (<0.1 mm), close (0.1–5 mm), and free (>5 mm).

Depth of invasion (DOI) was not analyzed because it was not consistently recorded before 2017, when it was first incorporated into the AJCC 8th edition; its exclusion prevented potential selection bias.

Tumor recurrence was detected using clinical examination, incisional biopsy or fine-needle aspiration cytology (FNAC), and radiological imaging (CT, MRI, PET-CT). Recurrence was defined as a lesion of similar histology arising at the same anatomical site ≥6 weeks and ≤5 years following primary surgery. Lesions occurring within 6 weeks were considered persistent disease and excluded. Local recurrence was defined as involvement of the primary surgical bed, and regional recurrence as disease in cervical lymph nodes. Distant metastases were excluded from analysis.

### 2.2. Treatment

All patients were diagnosed in our center with incisional biopsy. Preoperatory study included complete physical examination, facial and cervico-thoracic CT, cervico-facial MRI and blood test, and were presented at Head and Neck Tumor Board before treatment. All included patients underwent radical tumor resection as the primary treatment. Neck dissection was performed when indicated. Selective neck dissection (levels I–IV) was performed for clinically node-negative cases (cN0), and functional or modified radical dissection (levels I–V) for node-positive disease (cN+). Bilateral neck dissection was indicated for midline or bilateral tumors. Histopathological findings were reviewed by dedicated pathologists, and adjuvant therapy (radiation or chemoradiation) was administered according to margin status and adverse features.

Post-treatment follow-up consisted of monthly visits during the first 6 months, every 3 months up to 2 years, biannually until year 5, and annually thereafter.

### 2.3. Statistical Analysis

All statistical analyses were conducted using Python version 3.12.2. Descriptive statistics were used to summarize patient demographics and clinicopathological characteristics. Categorical variables were expressed as frequencies and percentages, and continuous variables as means ± standard deviation or medians [interquartile range], depending on data distribution.

Associations between clinicopathological variables and recurrence were assessed using the Chi-squared test for independence. When expected cell counts were less than five, Fisher’s exact test was used to ensure statistical validity. The primary study endpoint was recurrence-free survival (RFS), defined as the time from the date of primary surgery to the date of first documented locoregional recurrence or the last follow-up without recurrence. Survival distributions were estimated using the Kaplan–Meier method, and differences between groups were assessed using the log-rank test.

To identify predictors of recurrence, univariate Cox proportional hazards regression analysis was first performed for each clinicopathological variable. Variables with a significance level of *p* < 0.10 in univariate analysis were subsequently entered into a multivariate Cox proportional hazards model to identify independent predictors of recurrence.

Results were reported as hazard ratios (*HR*s) with 95% confidence intervals (CIs). Statistical significance was defined as a two-sided *p*-value < 0.05.

To visualize recurrence timing according to risk stratification groups, a violin plot combining kernel density estimation and boxplot statistics was generated. Median recurrence times and interquartile ranges were reported for each risk group. Only patients who developed recurrence were included in this analysis.

### 2.4. Nomogram Development and Recurrence Risk Score Calculation

Based on the independent predictors identified in the multivariate Cox model, a nomogram was developed to estimate individual probabilities of locoregional recurrence. Nomograms provide a graphical representation of a multivariate predictive model, allowing individualized risk estimation by assigning weighted points to each prognostic factor and summing these to obtain a total score corresponding to a predicted probability of outcome.

To facilitate clinical application, the regression model was translated into a simplified Recurrence Risk Score (RRS). The weighted contribution of each predictor was determined using the β-coefficients obtained from the multivariate Cox model, which represent the logarithmic transformation of the hazard ratios. To construct the scoring system, β-coefficients for each independent variable were scaled relative to the smallest coefficient in the model and subsequently rounded to the nearest integer. This process allowed the transformation of the continuous regression model into a practical point-based scoring system suitable for routine clinical use. Age, gender, alcohol and tobacco use were not included in the score calculation because they did not show a statistically significant association with locoregional recurrence in the multivariate Cox regression analysis.

The total RRS for each patient was calculated by summing the points assigned to each prognostic factor present. Based on the distribution of total scores and their association with recurrence outcomes, patients were stratified into three clinically meaningful risk categories: Low risk; Intermediate risk; High risk. These categories were subsequently used to evaluate recurrence incidence and recurrence-free survival across risk groups.

## 3. Results

### 3.1. Patient Characteristics

A total of 332 patients were included in this study, 185 male (56%) and 147 female (44%). The mean age at diagnosis was 64.3 ± 13.56 years. The tongue was the most common primary site (44.9%). Advanced tumor size (T3/T4) were diagnosed in 147 patients (44.27%). All patients underwent surgical treatment, with 134 patients (40.36%) receiving adjuvant radiotherapy (68 patients) or chemo-radiotherapy (66 patients). Neck dissection was performed in 264 patients (79.51%), and 101 (30.42%) had positive lymph node status (pN+). Extracapsular spread was present in 31 cases (9.33%) among those who underwent neck dissection. Free resection margins (R0 = > 5 mm) were achieved in 221 cases (66.56%), while close resection margins (R1) were observed in 105 patients (31.62%). In only 6 cases (1.80%), involved (positive) margins were reported. All patients were discussed in a multidisciplinary tumor board upon availability of final histopathological results, where decisions regarding adjuvant treatment were made based on margin status and other risk factors.

Among the 105 patients with close resection margins, 38 (36.19%) were managed with close surveillance, 30 (28.57%) underwent surgical re-excision for margin widening, and 37 (35.23%) received adjuvant chemoradiotherapy due to the presence of additional associated risk factors. Regarding the 6 patients with involved margins, surgical re-intervention with margin extension was feasible in 2 cases (33.33%). In the remaining 4 patients (66.66%), given the presence of locoregionally advanced disease, adjuvant treatment with chemoradiotherapy was indicated.

Histopathological grading was moderately and poorly differentiated (G2/G3) in the vast majority of the sample, present in 222 of the cases (66.86%). Comprehensive clinicopathological characteristics and their association with recurrence are summarized in Table 1.

### 3.2. Risk Factors in Association with Locoregional Recurrence

Univariate analysis confirmed a significant correlation between resection margin status (*p* = 0.000), T-stage (*p* = 0.005), lymph node status (*p* = 0.002), histopathological grading (*p* = 0.000), and extracapsular spread (*p* = 0.000) with recurrence risk.

**Figure 1:**
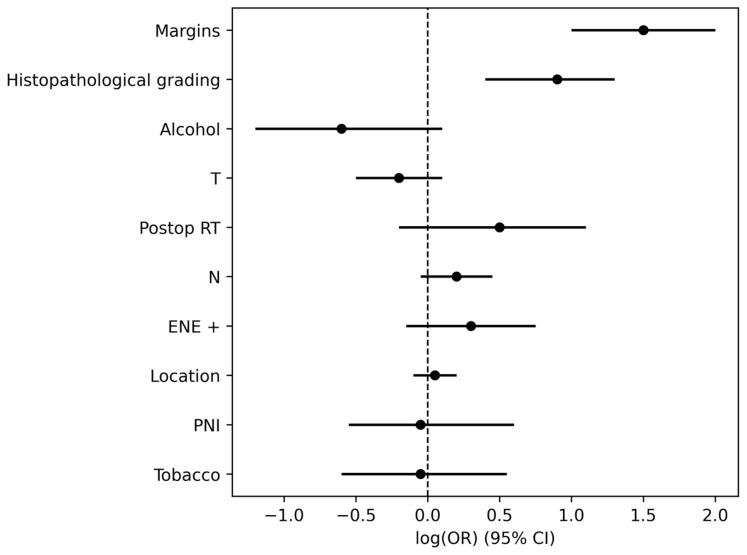
Forest plot showing hazard ratios (*HR*) and 95% confidence intervals for recurrence predictors in the multivariate Cox model. The vertical dashed line represents the null value (*HR* = 1.0). A significant association is observed for margin status (*HR* = 4.15) and Histopathological grading (*HR* = 2.34).

Multivariate Cox regression analysis identified close resection margins (*p* = 0.000) and advanced histopathological grading (*p* = 0.0012) as independent predictors of tumor recurrence. Results of univariate and multivariate analyses are summarized in Table 2, and their hazard ratios are illustrated in Figure 1.

### 3.3. Recurrence-Free Survival Analysis

**Figure 2:**
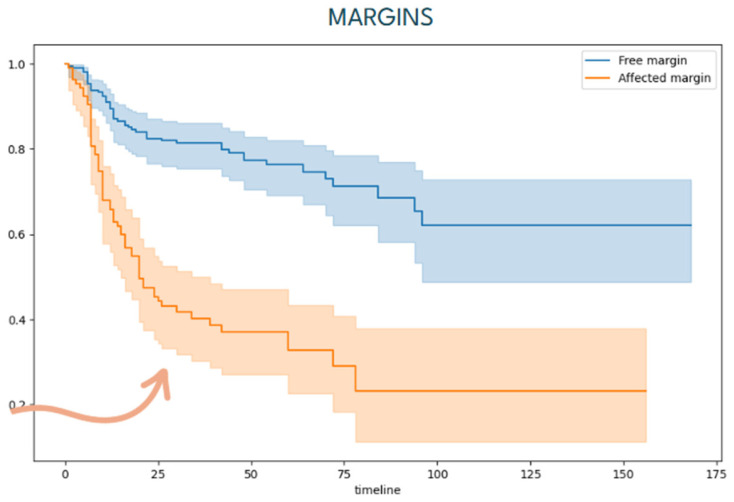
Kaplan–Meier disease-free survival curve stratified by proximal resection margin status. Patients with proximal (close or positive) resection margins experienced significantly earlier recurrence than those with wider/negative margins (univariate analysis). The arrow emphasizes that having affected margins is associated with significantly poorer early survival.

Among the 332 patients, the median follow-up time from surgery to the last contact with the patient was 58.16 ± 15.23 months (range 6 to 120 months). During this period, 115 patients (34.63%) developed locoregional recurrence, occurring at a mean of 19.94 ± 8.04 months following primary treatment. Disease-related mortality was observed in 77 out of 332 patients (23.19%). Free-disease survival analysis shows that most recurrences occurred during the first 24 months of follow-up (79.13%). Univariate analysis showed proximal resection margins were significantly correlated with earlier recurrence (Figure 2).

**Figure 3:**
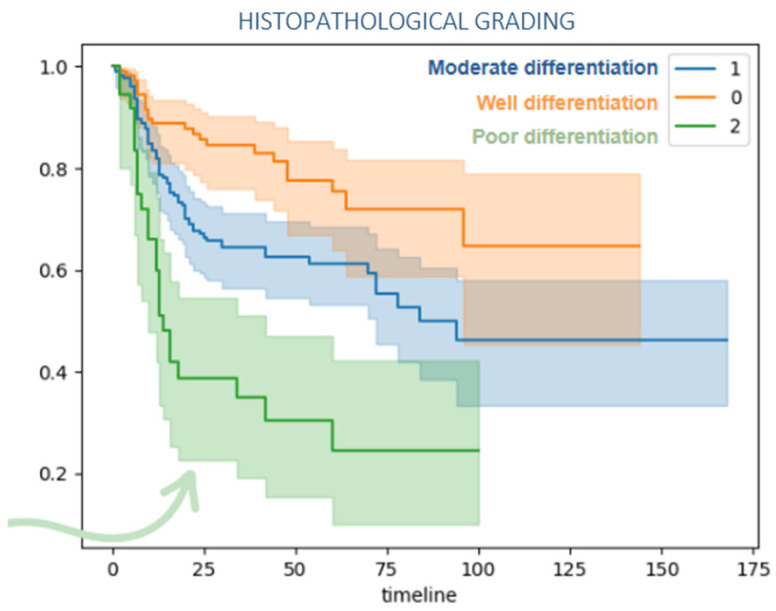
Kaplan–Meier disease-free survival curve by histopathological tumor grade. The arrow emphasizes that poorly differentiated tumors are associated with significantly reduced early survival, making it the most aggressive group in the chart.

Poorly differentiated tumors also presented higher risk of recurrence in shorter time compared to well and moderated differentiated, Figure 3.

**Figure 4:**
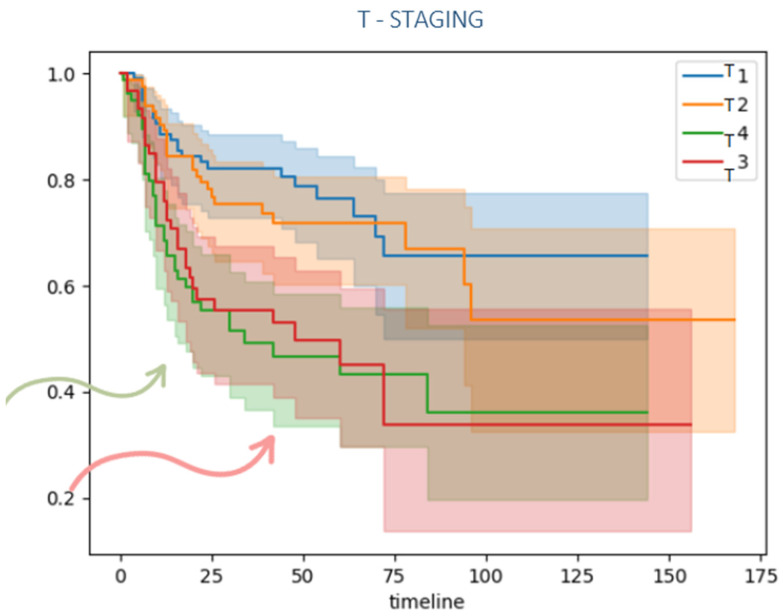
Kaplan–Meier disease-free survival curve comparing T stage. Patients with locally advanced primary tumors (T3–T4) exhibited a significantly lower disease-free survival than those with lower T stage. The arrows emphasize that higher T-stage is associated with earlier and more rapid decline in survival.

**Figure 5:**
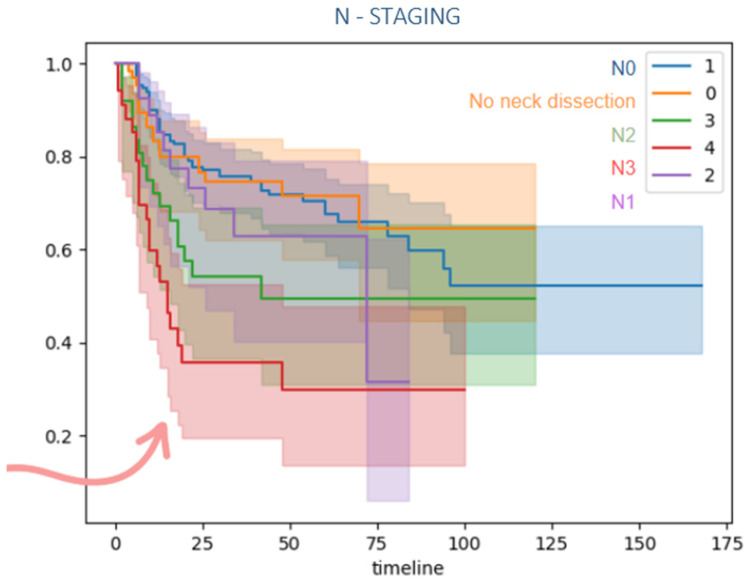
Kaplan–Meier disease-free survival curve comparing nodal status. Patients with lymph node metastasis (N+) demonstrated a significantly reduced disease-free survival compared with node-negative patients (N0). The arrow emphasizes that advanced nodal involvement (N3) is associated with markedly poorer early survival, making it the highest-risk group in this chart.

**Figure 6:**
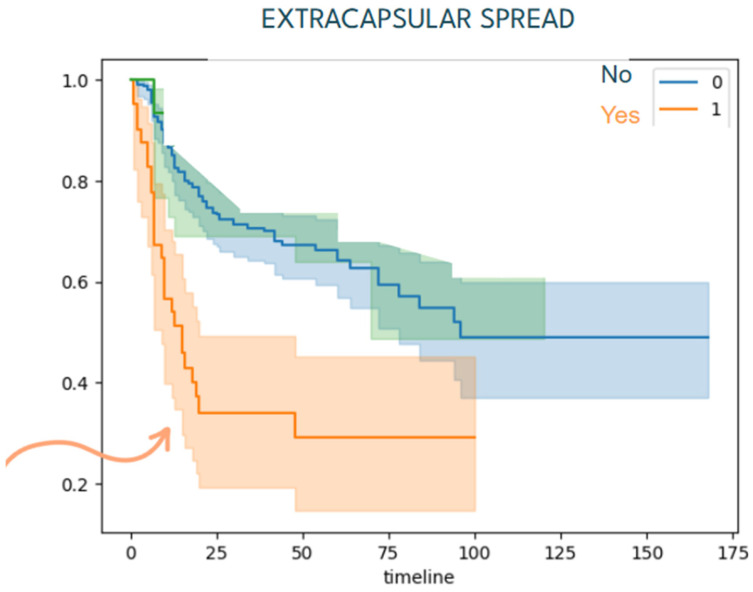
Kaplan–Meier disease-free survival curve by extracapsular spread (ECS) status. Patients exhibiting extracapsular spread of nodal disease experienced significantly worse disease-free survival than those without ECS. The arrow emphasizes that presence of extracapsular spread is associated with markedly poorer early survival.

Patients with local advanced disease (T3/T4) and lymph node metastasis also presented a lower disease-free survival compared to lower TNM classification as it is shown in Figure 4, Figure 5 and Figure 6.

The recurrence risk score (RRS) was developed based on the most significant predictors of recurrence identified through univariate and multivariate Cox regression analyses. Weighted points were assigned to each variable according to their hazard ratios in the multivariate model. The final scoring system included the following components (Table 3).

According to their total RRS, patients were stratified into three risk categories: low risk (0–3 points; 5-year recurrence-free survival > 75%), intermediate risk (4–6 points; 5-year recurrence-free survival 40–75%), and high risk (≥7 points; 5-year recurrence-free survival < 40%).

The RRS was calculated for all patients, classifying them into three risk groups based on total scores (Table 4):

- Low-risk group (0–3 points): 213 patients (64.16%)
- Intermediate-risk group (4–6 points): 91 patients (27.41%)
- High-risk group (≥7 points): 28 patients (8.43%)

**Figure 7:**
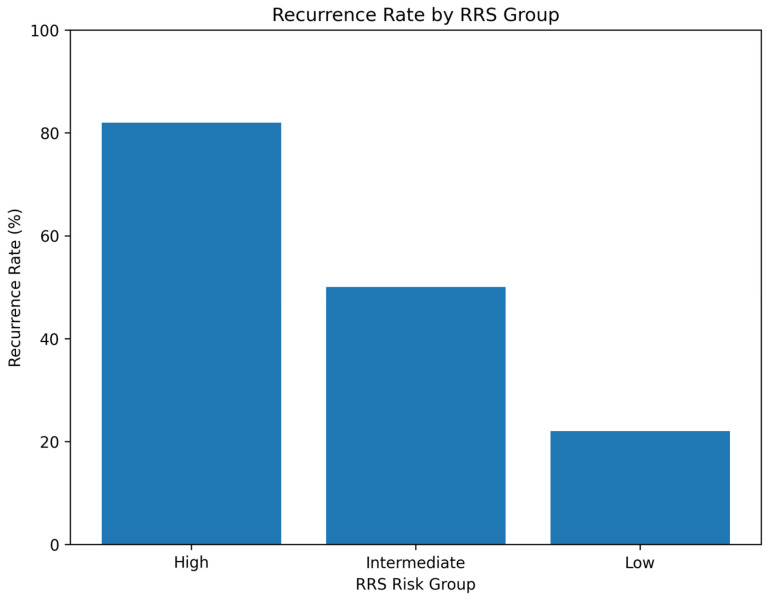
Recurrence rates stratified by RRS group. The bar chart shows the percentage of patients in each group who developed locoregional recurrence: 21.6% in the low-risk group, 50.6% in the intermediate-risk group, and 82.1% in the high-risk group (*p* < 0.001).

Recurrence analysis demonstrated significant differences among the three RRS groups (*p* < 0.001). Patients in the high-risk group (RRS ≥ 7) exhibited the highest recurrence rate (82.1%), while those in the intermediate-risk group (RRS 4–6) had a 50.6% recurrence rate, and the low-risk group (RRS 0–3) had the lowest recurrence rate (21.6%) (Figure 7).

Among the subset of patients who developed locoregional recurrence, time to first recurrence varied by RRS group.

**Figure 8:**
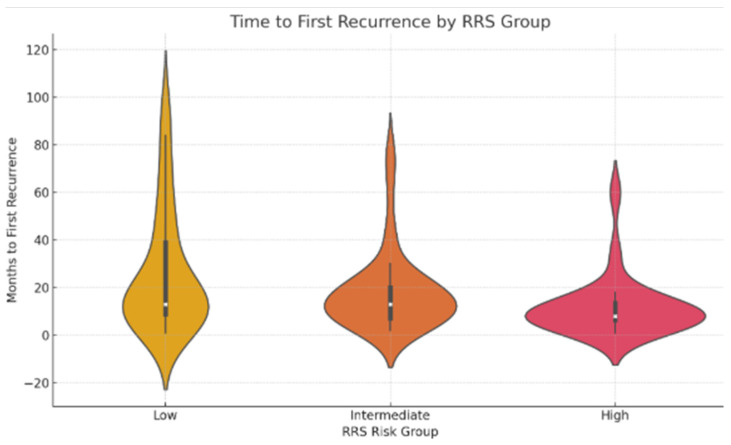
Violin plot showing the distribution of time to first recurrence across RRS groups. Narrower distributions and shorter median times were observed in higher-risk categories.

Patients in the high-risk group (RRS ≥ 7) experienced the earliest recurrences, with a median time to recurrence of 8.0 months (interquartile range [IQR]: 6.5–13.5 months). In the intermediate-risk group, the median time to recurrence was 13.0 months (IQR: 7.2–20.0 months). Similarly, patients in the low-risk group showed a median recurrence time of 13.0 months, but with a wider IQR (9.0–39.0 months), suggesting a more heterogeneous pattern of recurrence timing. The violin plot in Figure 8 illustrates progressively narrower distributions and earlier recurrence times as risk increased from low to high.

## 4. Discussion

Current management of OSCC involves a multidisciplinary team and, to date, wide surgical excision remains the gold-standard treatment, with adjuvant radiotherapy and/or chemotherapy recommended when high-risk clinicopathological factors are present. In this study, all 332 patients underwent surgical treatment as the primary treatment, with 134 (40.36%) receiving adjuvant therapy based on individualized risk assessment.

### 4.1. Patterns and Timing of Recurrence

There are no universally accepted criteria for defining tumor recurrence. Recurrence is generally considered the appearance of a tumor with similar histological characteristics in the same or nearby location, occurring between 6 weeks after surgery and within the first 3 years [11,12]. Tumor recurrence remains one of the primary determinants of mortality in patients with OSCC, with a 5-year survival rate of below 40% [6,13,14]. Despite aggressive multidisciplinary treatment strategies, recurrence rates of 15–40% are reported in the literature. In our cohort, 115 patients (34.63%) developed locoregional recurrence during a median follow-up of 58.16 months, with a mean time to recurrence of 19.94 ± 8.04 months, which is consistent with previously published data. This relatively high recurrence rate may be attributed to the tertiary care setting of our institution, where more complex oncological cases are referred. Notably, 79.13% of all recurrences occurred within the first 24 months after surgery, underscoring the importance of close early surveillance. This finding reinforces the concept of a “high-risk window” in the first two years post-treatment, during which patients require intensified surveillance, including imaging and clinical examination every 1–3 months, depending on risk stratification [15].

Local recurrence near the primary tumor location occurs in 8–19% of patients [5,16], in our sample, 17% of patients developed local recurrence (54% of all recurrences). Regional recurrences are described in 15–40% of cases [5,6,17]; in our study, 16.20% of patients developed regional recurrence (46% of all recurrences). We decided to combine local and regional recurrences into locoregional recurrence in order to have a higher statistical power as it avoids small-sized subgroup bias [6,18,19].

### 4.2. Independent Predictors of Recurrence

The present study analyzed a large cohort of surgically treated OSCC patients and identified key clinicopathological factors independently associated with locoregional recurrence. Close or positive resection margins and poor histological differentiation emerged as the most significant predictors of recurrence, consistent with prior literature [20,21]. The presence of ECS, in particular, has emerged as a marker of highly aggressive disease and is strongly associated with both local and distant metastasis [22].

Interestingly, while perineural invasion (PNI) and lymphovascular invasion (LVI) were not statistically significant predictors of recurrence in our multivariate model, numerous meta-analyses have reported their adverse prognostic impact [21].

Management of surgical margins remains a critical aspect of treatment planning. Achieving free margins greater than 5 mm is the primary goal, as this has been associated with improved local control and higher 5-year overall survival [20,21]. Margin status is also a key determinant in guiding the need for adjuvant therapies such as radiotherapy or chemotherapy. However, the definition of close surgical margins is not well established in the literature. An increased risk of local recurrence has been observed in patients with margins < 2.2 mm [23], and a better prognosis in those patients with margins > 5 mm.

Our results confirm that patients with close or positive margins exhibited a fourfold higher recurrence risk compared with those with free margins. These findings reinforce the need for meticulous surgical technique, intraoperative margin assessment, and consideration of re-resection when feasible.

Despite the low incidence of positive margins (1.80%), their presence remains clinically relevant. When feasible, re-resection is preferred [24]. However, anatomical constraints or patient comorbidities may limit surgical options. In these situations, combined adjuvant radiotherapy and chemotherapy have been associated with enhanced local control and reduced recurrence rates.

Histopathological differentiation is another well-established prognostic indicator [7]. Poorly differentiated tumors exhibit higher proliferative capacity, greater invasive potential, and higher likelihood of nodal metastasis, all of which contribute to increased recurrence risk [25]. Furthermore, this poor differentiation correlates with reduced sensitivity to treatment and a higher likelihood of relapse [21,26]. In our cohort, moderate or poor differentiation independently doubled the risk of locoregional relapse, highlighting its continued importance as a core variable in risk assessment models.

In addition to margin status and tumor differentiation, other well-established pathological variables demonstrated significant associations with recurrence in our cohort. Advanced primary tumor stage (T3–T4) was linked to a higher recurrence rate compared with early-stage disease (T1–T2), aligning with previous reports that correlate tumor size with increased local failure and poorer outcomes [8,27]. Likewise, nodal involvement (N+ status), observed in 30.4% of our patients, was strongly associated with higher recurrence risk and reduced survival, underscoring its central role as a prognostic factor in OSCC [28,29].

Extracapsular spread (ECS), identified in 14.5% of cases, was also significantly associated with regional recurrence and poorer survival, consistent with prior evidence highlighting ECS as an indicator of tumor aggressiveness and systemic dissemination [30,31]. Mermod et al. [32] reported a significant correlation between ECS and distant metastases, with an hazard ratio of 2.18.

Although perineural invasion (PNI) was identified in 43.7% of patients, it did not achieve statistical significance as a predictor of recurrence in our multivariate model (*p* > 0.05). Similar findings have been reported by Aivazian et al. [33], and others, suggesting that conventional binary classification of PNI (“present” vs. “absent”) may underestimate its prognostic impact [34]. Despite the lack of statistical significance in our multivariate model, the prognostic value of PNI in OSCC should not be disregarded. Numerous studies have emphasized its importance, especially in early-stage tumors where other high-risk features are absent [21,35,36]. The identification of PNI often indicates a more aggressive tumor phenotype and may necessitate more extensive surgical management, including neck dissection. Furthermore, Bakst et al. [37] suggest that the presence of extensive or large-caliber PNI should be considered an indication for adjuvant radiotherapy.

Interestingly, postoperative radiation therapy was associated with higher recurrence rates in univariate analysis (*p* < 0.001). This likely reflects selection bias, as adjuvant treatment is typically administered to patients with more advanced or high-risk disease. Consequently, this finding should not be interpreted as a causal relationship but rather as a reflection of underlying disease severity. This finding underscores the importance of optimizing adjuvant treatment protocols, particularly for patients with high RRS values (≥7), who may benefit most from intensified multimodal management.

In parallel with advances in predictive modeling, oncology research is also witnessing increasing interest in minimally invasive and technology-driven therapeutic approaches, as highlighted in recent studies exploring innovative energy-based systems in dermatologic and oncologic applications [38].

No significant correlation was observed between recurrence and age, sex, smoking or alcohol history, or tumor site, contrasting with some reports suggesting the lateral tongue and floor of mouth as higher-risk locations due to lymphatic drainage patterns [18].

Traditional staging systems provide a framework for prognostication but do not fully account for the multifactorial nature of tumor recurrence. In recent years, increasing attention has also been directed toward the role of systemic inflammatory biomarkers in the prognostic stratification of OSCC. For instance, Nicoară et al. [39] reported that indices derived from routine hematological parameters, such as the systemic immune–inflammation index (SII) and neutrophil-to-lymphocyte ratio (NLR), may provide valuable information regarding the host immune response and treatment-related inflammatory changes during OSCC management. These findings suggest that inflammatory biomarkers may complement conventional clinicopathological variables in predicting tumor behavior and treatment outcomes.

In parallel, growing evidence highlights the importance of tumor–microenvironment interactions and immune signaling pathways in the progression and recurrence of head and neck squamous cell carcinoma. Alterations in the tumor microenvironment, including immune cell infiltration, inflammatory signaling, and stromal remodeling, have been shown to influence tumor aggressiveness, therapeutic resistance, and the likelihood of recurrence. These biological mechanisms indicate that recurrence risk is not determined solely by traditional clinicopathological parameters but is also shaped by complex interactions between tumor cells and the surrounding microenvironment [40].

In this context, clinicopathological prediction models such as the Recurrence Risk Score (RRS) may represent an initial step toward integrated prognostic systems that combine conventional pathological variables with emerging molecular and immunological biomarkers.

Consequently, models that integrate several independent predictors into a single composite score offer greater predictive accuracy. Nomograms have gained wide acceptance in oncology, as they translate multivariable statistical models into clinically interpretable tools for individualized risk prediction [9,10]. Based on the findings of our study, we developed a nomogram based RRS that effectively stratifies patients into distinct risk categories, providing a practical and individualized prognostic tool for clinical use.

The RRS stratified patients into low-, intermediate-, and high-risk categories, with recurrence rates of 21.6%, 50.6%, and 82.1%, respectively. Importantly, high-risk patients demonstrated both a greater incidence and earlier onset of recurrence, emphasizing the model’s capacity to identify individuals requiring intensified follow-up or adjuvant therapy.

From a clinical standpoint, the RRS could support risk-adapted surveillance protocols, guiding clinicians to allocate resources more effectively. For example, patients in the high-risk group may benefit from shorter follow-up intervals, early adjuvant therapy consideration, or inclusion in clinical trials for intensified treatment regimens. Conversely, low-risk patients may avoid unnecessary interventions, minimizing treatment-related morbidity and improving quality of life.

This study has several strengths. It includes a relatively large, homogeneous cohort treated within a single tertiary referral center with consistent surgical and histopathological protocols. Moreover, the use of based statistical modeling ensured analytical transparency and reproducibility.

However, several limitations should be acknowledged. First, the retrospective design of the study may introduce inherent biases related to data collection and patient selection. In addition, the single-center nature of the present study may limit the generalizability of the findings, as treatment protocols, surgical practices, and patient characteristics may vary across institutions. Consequently, the predictive performance of the proposed RRS may differ in external populations. Multicenter studies including larger and more heterogeneous patient cohorts will be essential to confirm the robustness, reproducibility, and external validity of the model. In addition, the RRS was derived from the present dataset and has not yet undergone external validation. Prospective validation in independent cohorts will therefore be essential before its widespread clinical implementation. Although the present study developed a nomogram-derived recurrence risk score, internal validation techniques such as bootstrap resampling or cross-validation were not performed. Future studies should incorporate these validation approaches to assess model stability and reduce the risk of overfitting before clinical implementation. In addition, performance metrics commonly used for prognostic models, such as the concordance index (C-index) and calibration plots, were not evaluated in the present study. Future research should include these discrimination and calibration analyses to more precisely assess the predictive accuracy and clinical applicability of the proposed recurrence risk model. Additionally, the model relies solely on clinicopathological factors, overlooking emerging biomarkers such as HPV status or gene expression profiles, which could enhance the accuracy of risk prediction. Another important limitation is that DOI was not included in the present analysis because this parameter was not consistently reported in pathology records before the implementation of the AJCC 8th edition staging system in 2017. Including DOI would therefore have resulted in significant data loss and potential selection bias. Nevertheless, DOI is a well-recognized prognostic factor in oral squamous cell carcinoma, and future studies evaluating similar recurrence risk models should incorporate this variable when complete datasets are available. Despite using multivariate Cox regression, potential confounding variables may still influence recurrence risk. Variability in adjuvant therapy regimens across patients could also affect recurrence risk, introducing potential bias in the risk score.

From a clinical perspective, the development of accessible prognostic tools such as the RRS may help clinicians identify patients at higher risk of locoregional relapse following surgical treatment of OSCC. Risk-adapted models could support more individualized postoperative management, guiding decisions regarding follow-up intensity, imaging surveillance, and consideration of adjuvant therapies. In the future, integrating clinicopathological models such as the RRS with emerging molecular and immunological biomarkers may further improve risk stratification and contribute to more personalized therapeutic strategies for patients with oral squamous cell carcinoma.

## 5. Conclusions

This study contributes to a deeper understanding of locoregional recurrence in OSCC by identifying key prognostic factors associated with disease relapse. Despite its retrospective nature, our findings highlight the significant role of margin status, tumor size, lymph node involvement, and histopathological grading in predicting recurrence.

A nomogram-based Recurrence Risk Score (RRS) was developed demonstrating good discrimination and calibration in predicting recurrence-free survival.

The RRS effectively stratifies patients into clinically meaningful risk groups, providing a practical, individualized tool for recurrence risk estimation.

Implementation of the RRS in clinical practice may enable personalized follow-up strategies and better selection of candidates for adjuvant therapy, optimizing long-term outcomes.

Further prospective, multicentric validation is warranted to confirm its external applicability and predictive robustness.

## Figures and Tables

**Table 1:** Clinicopathological characteristics of 332 patients with OSCC and their association with locoregional recurrence. Data are presented as number (percentage) of patients within each variable category and corresponding recurrence frequency.

Variable	Number (n = 332)	Number of Loccorregional Recurrence (n = 115)
Gender Male Female	185 (56%) 147 (44%)	57 (30.81%) 58 (39.45%)
Smoking Yes No	220 (66.26%) 112 (33.73%)	77 (35%) 38 (33.92%)
Alcohol Yes No	62 (18.67%) 270 (81.32%)	19 (30.64%) 96 (35.55%)
Location Tongue Alveolar ridge (mandible) Alveolar ridge (maxilla) Retromolar trigone Floor of the mouth Cheek Palate (hard and soft)	149 (44.87%) 58 (17.46%) 31 (9.33%) 29 (8.73%) 30 (9.03%) 31 (9.33%) 4 (1.20%)	48 (32.21%) 24 (41.37%) 14 (45.16%) 9 (31.03%) 7 (23.33%) 11 (35.48%) 2 (50%)
T—Classification T1 T2 T3 T4	98 (29.5%) 87 (26.2%) 62 (18.7%) 85 (25.6%)	23 (23.46%) 25 (28.78%) 29 (46.77%) 38 (44.70%)
N—Classification N0 N1 N2 N3	231 (69.57%) 28 (8.43%) 38 (11.44%) 35 (10.54%)	66 (55.91%) 11 (39.28%) 17 (44.73%) 21 (60%)
Tumor margins Free (>5 mm) Close (<5 mm) Positive	221 (66.56%) 105 (31.62%) 6 (1.80%)	50 (22.62%) 61 (58.09%) 4 (66.66%)
Grading Well differentiated Moderately differentiated Poorly differentiated	110 (33.13%) 183 (55.12%) 39 (11.74%)	23 (20.09%) 68 (37.15%) 24 (61.58%)
Extracapsular node spread No Yes	284 (85.54%) 48 (14.45%)	99 (34.85%) 16 (33.33%)
Perineural invasion Yes No	145 (43.67%) 187 (56.32%)	56 (38.62%) 59 (31.55%)
Postoperative radiation Yes No	134 (40.36%) 198 (59.63%)	65 (48.50%) 50 (25.25%)

**Table 2:** Univariate and multivariate Cox regression analyses for factors associated with locoregional recurrence. *HR*: Hazard ratio. PNI: Perineural invasion. RT: Postoperative Radiotherapy. ECS: Extracapsular spread.

Variable	Univariate		Multivariate	
	*HR* (95%)	*p*	*HR* (95%)	*p*
Alcohol	0.8 [0.44–1.45]	0.5587	0.56 [0.27–1.13]	0.1114
Smoker	1.05 [0.65–1.69]	0.9426	0.94 [0.53–1.67]	0.8399
Location	0.6 [0.53–0.73]	0.4993	0.89 [0.76–0.95]	0.7232
PNI	0.73 [0.46–1.15]	0.2200	0.96 [0.56–1.62]	0.8657
RT	1.59 [1.45–1.71]	**0.000**	0.95 [0.64–1.39]	0.172
ECS	2.79 [1.75–4.45]	**0.0001**	1.31 [0.86–2.01]	0.212
T-classification	1.43 [1.17–1.75]	**0.0022**	0.8 [0.6–1.09]	0.1554
N-classification	1.43 [1.19–1.72]	**0.0032**	1.22 [0.95–1.57]	0.1196
Margin status	4.25 [2.66–6.78]	**0.000**	4.15 [2.37–7.39]	**0.000**
Histopathological Grading	2.04 [1.64–3.45]	**0.000**	2.34 [1.46–3.74]	**0.0004**

**Table 3:** This table summarizes the variables included in the recurrence risk score and their corresponding point assignments based on multivariate Cox regression analysis. Factors include resection margin status, histopathological grading, T-classification, lymph node status (N-classification), and extracapsular spread, with higher total scores indicating increased risk of recurrence.

Variable	Category	Points
Resection margin status	Free margins (R0)	0
	Close margins (R1)	3
	Positive margins (R2)	6
Histopathological grading	Well differentiated (G1)	0
	Moderately differentiated (G2)	1
	Poorly differentiated (G3)	3
T-classification	T1–T2	0
	T3–T4	1
Lymph node status (N-classification)	N0	0
	N+ (N1–N3)	1
Extracapsular spread	Absent	0
	Present	1

**Table 4:** Recurrence rates stratified by risk group based on RRS. Data show the number and percentage of patients without and with recurrence within each risk category.

Risk Group	Total Patients	No Recurrence	No Recurrence Rate (%)	Recurrences	Recurrence Rate (%)
High risk (≥7 points)	28	5	17.66%	23	82.14%
Intermediate-risk (4–6 points):	91	45	49.5%	46	50.55%
Low-risk (0–3 points):	213	167	78.4%	46	21.6%

## Data Availability

The original contributions presented in this study are included in the article. Further inquiries can be directed to the corresponding authors.
